# Novel potential tumor biomarkers: Circular RNAs and exosomal circular RNAs in gastrointestinal malignancies

**DOI:** 10.1002/jcla.23359

**Published:** 2020-05-17

**Authors:** Yezhao Wang, Zhe Li, Suyuan Xu, Junming Guo

**Affiliations:** ^1^ Department of Biochemistry and Molecular Biology, and Zhejiang Key Laboratory of Pathophysiology Ningbo University School of Medicine Ningbo China

**Keywords:** biomarkers, circular RNAs, exosome, gastrointestinal malignancies, non‐invasive diagnosis

## Abstract

**Background:**

Circular RNAs (circRNAs) are structural ubiquitous RNA molecules. Accumulating evidences have elucidated that circRNAs play essential roles in the pathogenesis of diseases including cancers. Exosomal circRNAs are those circRNAs stably existing in exosomes and having high clinical values as novel potential diagnostic biomarkers of many diseases. Gastrointestinal (GI) malignancies, including pancreatic cancer, colorectal cancer, hepatocellular carcinoma (HCC), and gastric cancer, are leading causes of mortality worldwide and a major global health burden. However, no ideal tumor biomarkers of screening early GI cancers are currently available.

**Methods:**

We collected data through Web of Science. The search terms used were as follows: circular RNA, circRNA, exosomes, exosomal circRNAs, biomarkers, gastrointestinal malignancies, pancreatic cancer, hepatocellular carcinoma, HCC, gastric cancer, colorectal cancer, physiological functions, biogenesis, molecular mechanism. Only articles published in English were included.

**Results:**

We found that several circRNAs and exosomal circRNAs have been used as potential biomarkers to screen GI cancers including pancreatic cancer (hsa_circ_0001649, circ_0007534, circ_0030235, circRHOT1, circZMYM2, circ‐LDLRAD3, chr14:101402109‐101464448C, chr4:52729603‐52780244C, circ‐IARS, and circ‐PDE8A), HCC (circSETD3, circADAMTS13, hsa_circ_0007874, hsa_circ_104135, circFBLIM1, cSMARCA5, circRNA‐100338, and circPTGR1), colorectal cancer (hsa_circ_0001178, hsa_circ_0000826, hsa_circ_0004771, circDDX17, circITGA7, and circHIPK3), and gastric cancer (hsa_circ_0074362, circNRIP1, circAKT3, circ‐DONSON, circPSMC3, circ‐KIAA1244, circPVRL3, circPVT1, hsa_circ_0000096, ciRS‐133, hsa_circ_0001017, and hsa_circ_0061276).

**Conclusion:**

CircRNAs and exosomal circRNAs have the potential high clinical diagnostic values for GI malignancies.

## 1 INTRODUCTION

In the recent few decades, with the accomplishment of Human Genome Project, approximately 20 000 genes encoding proteins have been identified.^1^ However, only lower than 2% of genome actually codes proteins. The vast majority of genome, which accounts for almost 98%, is transcribed to non‐coding RNAs (ncRNAs).^2^ Compared with microRNAs (miRNAs) and traditional linear RNAs, circular RNAs (circRNAs) resist to ribonuclease R.^3^ Because of their covalently closed structure, circRNAs are stable and present in high abundance in blood.^4^, ^5^ CircRNAs are now considered as potential biomarkers in gastrointestinal (GI) malignancies.^6^, ^7^, ^8^ Thanks to the advancement of high‐throughput functional genomic screening biotechnology, an increasing number of researches have confirmed tumor‐associated circRNAs.^6^, ^9^

Exosomes are vesicles generated and released by cells. Exosomes, carrying a cargo of lipids, genetic materials, proteins, and their derivatives, modulate cells' behaviors, influence the extracellular system, and may be a source of human disease biomarkers.^10^, ^11^ Exosomal circRNAs (exo‐circRNAs) have unique profiles reflecting the characteristics of tumors.^10^ They would be new GI cancers biomarkers.

Gastrointestinal malignancies, which mainly include gastric cancer (GC), colorectal cancer (CRC), hepatocellular carcinoma (HCC), and pancreatic cancer, are main causes of mortality worldwide and a global burden. For example, pancreatic cancer with only approximately 5% patients surviving over 5 years is the deadliest cancer; CRC is the third most frequent tumor and the second major origin of deaths related to cancers in the USA; HCC is the fourth major origin of cancer‐associated death worldwide; GC is the fifth most frequent tumor and the third major burden associated with cancers in modern society.^12^ Nevertheless, most of patients with GI malignancies miss the therapeutic window.^12^ When found early, GI malignancies are highly curable. However, the early diagnosis of GI malignancies continues to be a major challenge.

Recent years, researchers have gradually realized that the developments of new technology for non‐invasive early detection are likely to be the most effective method for cutting down mortality of GI malignancies.

Commonly used biomarkers such as carbohydrate antigen 125 (CA125), CA 19‐9, squamous cell carcinoma antigen (SCCA), α fetoprotein (AFP), tissue inhibitor of metalloproteinases 1 (TIMP‐1), cytokeratin 19 fragments (CyFra21‐1), and carcinoembryonic antigen (CEA) could be found not only in serum of patients with GI malignancies but also in serum of healthy individuals.^13^ For example, the specificity and sensitivity of CEA to detect GC were 0.686 and 0.593, respectively, and those for CA19‐9 were 0.605 and 0.559, respectively.^14^ Obviously, these biomarkers interfere the diagnostic accuracy and sensitivity.

CircRNAs and exo‐circRNAs extracted from plasma are high‐efficient blood‐based biomarkers with great clinical significance. In recent years, with dramatic successes of considerable technological advances and the progression of techniques, such as real‐time and high‐sensitivity liquid biopsy assays, sensitive sequencing, high‐depth targeted next‐generation sequencing, sequencing somatic mutations, polymerase chain reaction (PCR), next‐generation sequencing, and droplet digital PCR (ddPCR) analysis, circRNAs and exo‐circRNAs have appeared potential high clinical values and could be used to detect various GI malignancies.^15^, ^16^ Compared with lower specificity and sensitivity of CEA and CA19‐9 to detect GC, we explored that the specificity and sensitivity of hsa_circ_0001017 in blood reached 0.794 and 0.811, respectively.^4^ Therefore, there is a great hope and promising broad clinical applications in screening GI malignancies through circRNA‐based approaches.

This review summarizes GI cancer‐associated circRNAs and exo‐circRNAs and their possible application in the diagnosis of GI cancers.

## 2 circRNAs AND EXOSOMES

### 2.1 The history of circRNA research

CircRNAs were first discovered in viruses 41 years ago.^17^ For decades, researchers considered circRNAs as products of splicing errors. Later, in 1996, circRNAs were described as self‐replicating molecules in rodent and human cancer cells.^18^ Nonetheless, circRNAs have been re‐evaluated mainly owing to the great advances in deep sequencing and computational approaches.^19^

### 2.2 The biogenesis and physiological functions of circRNAs

Nowadays, a major group of circRNAs have been reported to be generated by different mechanisms.^9^, ^19^ Most circRNAs are arisen from precursor mRNAs (pre‐mRNAs) through “out‐of‐order” splicing (a process called back‐splicing).^9^, ^20^ Multiple known circRNAs are from exons of protein‐encoding genes.^19^ circRNAs are crucial players involved in numerous biological processes, such as sponging of miRNAs, regulating parental gene transcription and tumorigenicity, protein kinase activation, angiogenic sprouting, generation of short proteins, and post‐transcriptional regulation in the pathogenesis of malignancies.^20^, ^21^, ^22^, ^23^, ^24^ Additionally, recent functional studies have reported many tissue‐specific and cell‐specific circRNAs, which were functionally characterized as efficient biomarkers in human diseases.^25^ For example, ciRS‐7 (also termed CDR1as) has been demonstrated to be a risk factor through sponging corresponding miRNAs.^26^, ^27^ CircSETD3 (hsa_circ_0000567) and circADAMTS13 also act as sponges of miRNAs and participate in HCC tumorigenesis.^28^, ^29^ Strikingly, hundreds of circRNAs including ciRS‐7 are highly abundant in the mammalian brains.^30^ Some researchers believe that ciRS‐7 could be possibly used as therapeutic target agents against Alzheimer's neuronal injury.^31^ Moreover, several studies showed that the expression of circRNAs elucidated various cardiovascular and cerebrovascular diseases.^32^, ^33^ Furthermore, some circRNAs may be potential therapeutic targets of gestational diabetes mellitus.^34^ Similarly, circHIPK3 was identified as an applicable therapeutic intervention for diabetic proliferative retinopathy.^35^

### 2.3 The functions of exosomes

Exosomes (diameter, 30‐160 nm) act on many biological processes and participate in substance delivery and signal transduction.^36^ Exosome formation occurs spontaneously within the endosomal network via re‐routing of multivesicles and are released through exocytosis.^37^ Trams et al described the concept of exosomes as microvesicles composed of plasma membrane in 1981.^38^ In 1985, Pan et al confirmed the presence of exosomes inside multivesicular endocytic compartments using immunoelectron microscopy.^39^ Nonetheless, the official name of these structures as “exosomes” was provided by Johnstone et al in 1987.^40^ Several recent dynamic light scattering, transmission, and atomic force microscopy studies have helped us address the exosomes function sites.^41^, ^42^ Several groups have performed diverse studies on potential biological functions of exosomes in medicine.^43^, ^44^ For instance, an exosome‐based drug delivery system with high application potential in cancer targeted therapy was reported.^43^ Additionally, cancer‐derived exosomes show a great potential for acting as biomarkers.^45^ These studies have provided new ideas about how exosomes may be used to treat cancers efficiently and effectively. However, exosomes may present as two‐edged sword. For instance, researchers found that exosomes play critical roles in neurological disorders and could spread pathological proteins in these neuropsychiatric deficits, such as ischemic stroke, Parkinson neurodegeneration, brain infarction, and Alzheimer's neuronal injury.^46^ Unfortunately, some tumor‐derived exosomes may promote cancer progression and accelerate metastasis by modulating the primary site of tumors and assisting metastatic cancer cells escape from immunologic surveillance.^47^

## 3 circRNAs AS BIOMARKERS IN GI MALIGNANCIES

**FIGURE 1:**
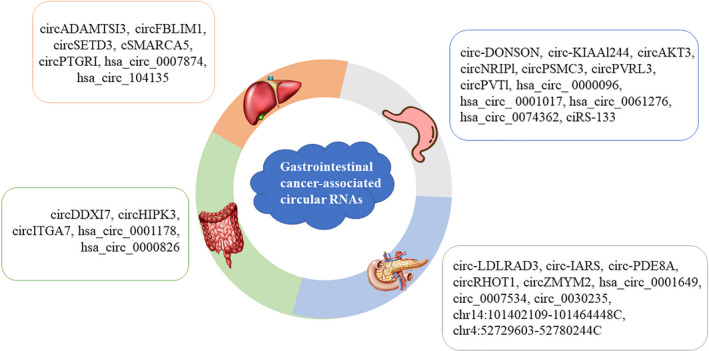
Gastrointestinal cancer‐associated circular RNAs

**FIGURE 2:**
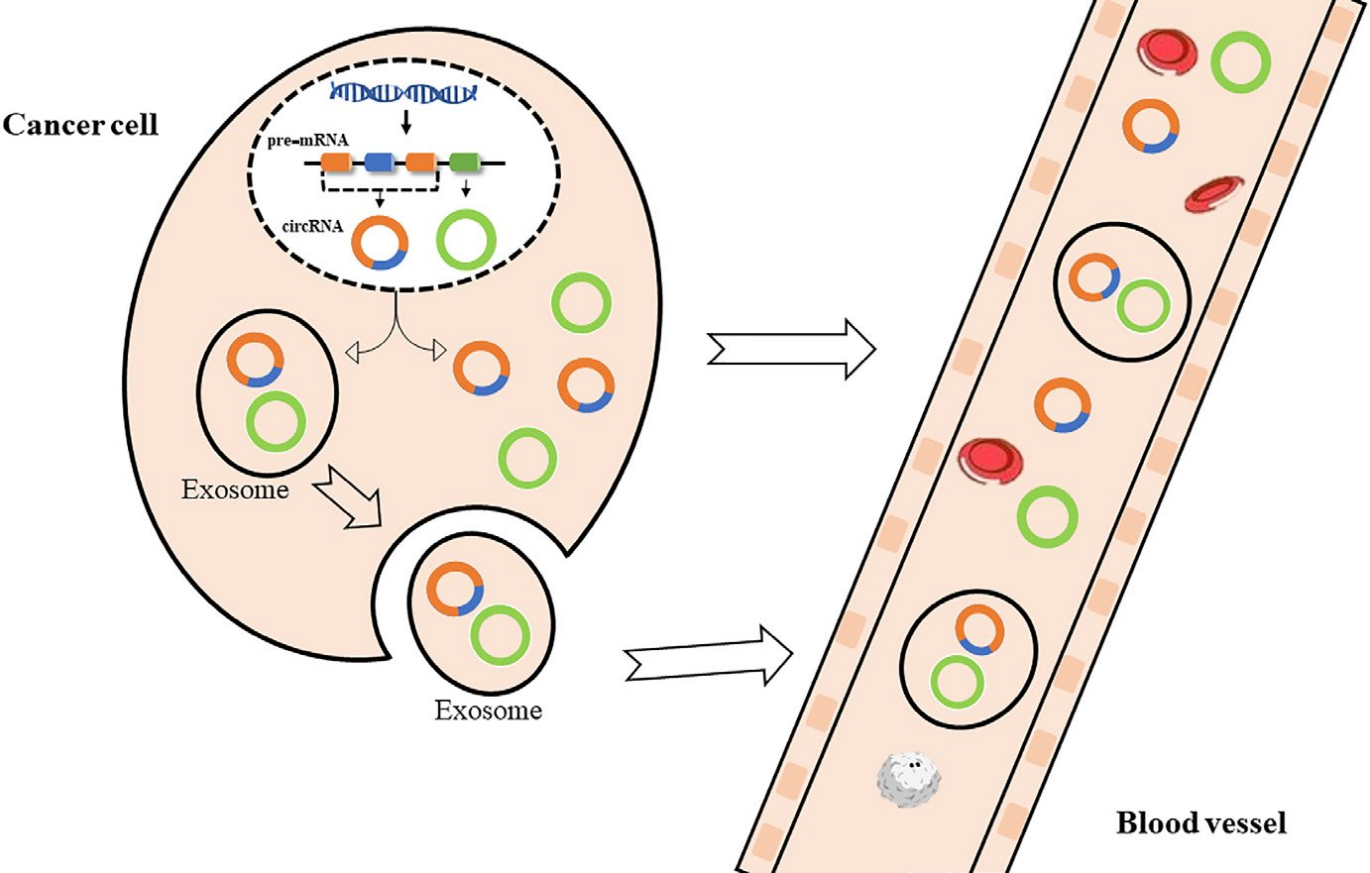
CircRNAs and exosomal circRNAs used as biomarkers. In the nuclei of cancer cells, circRNAs are produced by pre‐mRNA after transcription. The formation of circRNAs may involve one exon, several exons, several introns, or only one intron. CircRNAs may be released into the blood directly or with exosomes

Most advanced GI malignancies and their metastases are difficult to be cured. In contrast, patients with early GI cancer can be treated effectively. However, the main clinical imaging equipments such as digital X‐ray radiography, magnetic resonance imaging (MRI), and positron emission tomography‐computed tomography (PET‐CT) are not only expensive but also often quite subjective. Therefore, circRNAs and exo‐circRNAs as potential effective biomarkers may provide opportunities to properly treat patients with GI malignancies (Figure 1). CircRNAs are not easily degraded by enzymes such as exonucleases and ribonucleases. They have a greater stability and a longer half‐life in body fluids. Additionally, the expression of circRNAs in fluids and tissues often provides relatively high sensitivity and specificity in different developmental stages of GI malignancy patients. Therefore, circRNAs and exo‐circRNAs may be applied as biomarkers for GI cancer patients (Figure 2).

### 3.1 circRNAs as biomarkers of gastric cancer

Gastric cancer is a main origin of death in the modern society.^48^ Recent years, researchers have investigated circRNAs as biomarkers of GC. Based on RNA sequencing biotechnology, circNRIP1 and circAKT3 (hsa_circ_0000199) were explored to be increasingly expressed in human GC tissues; the increments of circNRIP1 and circAKT3 in human GC tissues were related to the metastasis potential and recurrence risk.^49^, ^50^ After purifying exosomes, Zhang et al found upregulated expression of circNRIP1 in GC patients.^49^ Their findings suggested that high levels of circNRIP1 indicated reduced survival.^49^ Huang et al found that the upregulation of circAKT3 in GC patients elucidated reduced five‐year disease‐free survival (DFS).^50^ The area under the curve (AUC) is 0.91, which elucidated that circAKT3 could be biomarker to predict survival.^50^ In addition, circ‐DONSON (hsa_circ_0004339) was markedly increased in GC tissues.^51^ The expression of circ‐DONSON was related to DFS, confirming circ‐DONSON as a novel GC biomarker.^51^

In contrast, the expression of circPSMC3 and circ‐KIAA1244 was significantly reduced in GC patients' plasmas.^52^, ^53^ Mechanism study elucidated that circPSMC3 might regulate the tumor progression through sponging miR‐296‐5p and might be applied as a potential therapeutic target of GC.^52^ Sun et al found that the attenuated expression of circPVRL3 could boost GC cells growth.^54^

Our group measured the expression profiles of circRNAs in cancer plasma from GC patients.^4^, ^6^ Aberrant expression of 308 circRNAs was demonstrated in GC tissues.^6^ Total 343 circRNAs in plasma from GC patients were further found.^4^ The above studies elucidated that circRNAs have advantages on the potential novel biomarkers of GC.

### 3.2 circRNAs as biomarkers of hepatocellular carcinoma

Currently, the incidence of HCC is supreme in Central America.^55^ Thousands of patients with HCC were diagnosed at late stages and lost the best opportunity of a surgical cure. Reduced circMTO1 expression in HCC was related to irreversible miss of therapeutic window for HCC patients.^56^ Bai et al discovered that silencing of circFBLIM1 could markedly inhibit HCC cells by directly sponging miR‐346.^57^ Additionally, underlying mechanism experiments showed that circSETD3 suppresses tumor growth by activating the cascade pathways.^29^ Furthermore, cSMARCA5 is known to act an essential role in the tumorigenesis of HCC.^58^ Our group found that hsa_circ_0068669 expression elucidated stages of HCC patients.^8^ These studies helped us uncover the great clinical value of circRNAs as biomarkers of HCC.

### 3.3 circRNAs as biomarkers of colorectal cancer

Colorectal cancer is common malignancies and the second cancer killer in the USA.^12^ Increasing the screening rate of CRC among people and finding effective treatment targets of CRC is imperative. Recently, scholars have identified circRNAs as effective biomarkers of CRC. Known to be associated with unfavorable clinicopathological factors, hsa_circ_0001178 and hsa_circ_0000826 clearly upregulated in CRC mucosae compared with adjacent normal mucosae.^59^ The AUCs were 0.816 and 0.945 for hsa_circ_0000826 and hsa_circ_0001178, respectively.^59^ Moreover, circDDX17 (hsa_circ_0002211) expression was lower in the CRC mucosae; and knockdown of circDDX17 could accelerate CRC cell growth and differentiation as well as suppress apoptosis.^60^ These results suggest that circDDX17 could serve as a tumor suppressor. Li et al further found that reduced circDDX17 expression was related to distant stage of CRC.^60^

Additionally, circITGA7 markedly inhibited CRC tumorigenesis.^61^ Interestingly, the level of circHIPK3 was notably higher.^62^ Moreover, increased expression of circHIPK3 was related to CRC distant metastasis, T status of tumor, and advanced clinical stage.^62^ These findings highlight circRNAs as biomarkers for CRC.

### 3.4 circRNAs as biomarkers of pancreatic cancer

Pancreatic cancer is largely incurable cancer with the lowest survival in the worldwide.^12^, ^63^ This challenge has attracted the interest of researchers, who have investigated circRNAs as new biomarkers of pancreatic cancer. Recently, two distinctly expressed circRNAs (chr14:101402109‐101464448C and chr4:52729603‐52780244C) were demonstrated to be novel potential biomarkers and drug targets of pancreatic ductal adenocarcinoma (PDAC).^64^ Circ_0007534 and circ_0030235 were markedly upregulated in PDAC; the expression of these circRNAs elucidated the poor prognosis of PDAC patients.^65^, ^66^ Furthermore, the expression of hsa_circ_0007534 indicated lymph node invasion.^65^ Xu et al found that positive lymph node invasion and higher tumor stage were associated with high expression of hsa_circ_0030235.^66^

CircRHOT1 (hsa_circ_0005397) and circ‐LDLRAD3 were also overexpressed in pancreatic cancer cells.^67^, ^68^ The circ‐LDLRAD3 in plasmas of PDAC patients was associated with stages.^68^ In addition, hsa_circ_0001649 was significantly downregulated in cells and tissue specimens of PDAC.^69^ Jiang et al confirmed that the low expression of hsa_circ_0001649 elucidated irreversible differentiation grade and stage through Fisher's exact tests.^69^ Jiang et al found that high hsa_circ_0001649 expression elucidated high overall survival (OS) rate.^69^ These studies verified that hsa_circ_0001649 could be applied as a biomarker of PDAC.

## 4 EXOSOMAL circRNAs AS BIOMARKERS OF GI MALIGNANCIES

Exosomes have been the increasingly interesting particles in medicine and biotechnology, especially for using as biomarkers in clinical diagnosis.^37^, ^70^ Their molecular constituents such as proteins, miRNAs, and circRNAs are stable and promising as diagnostic biomarkers in GI cancers.^71^, ^72^ Multiple previous studies have made the point that numerous tumor‐derived or tumor‐associated exo‐circRNAs could be reliable tumor‐specific biomarkers of GI cancers.^73^, ^74^, ^75^, ^76^, ^77^, ^78^, ^79^ For instance, circulating exosomal ciRS‐133 (hsa_circ_0010522) extracted from GC patients' serum and plasma samples was markedly higher compared to normal subjects.^73^ Wang et al also investigated that the expression levels of circPTGR1 (hsa_circ_0008043) in HCC patients' serum exosomes were clearly upregulated.^74^ Li et al discovered that the expression levels of exosomal circRNA IARS (circ‐IARS) in pancreatic cancer tissues and plasma specimens were significantly higher than healthy controls.^75^ Additionally, circulating exosomal circRNAs PDE8A (circ‐PDE8A) derived from PDAC patients' plasma exosomes was also abnormally overexpressed in pancreatic cancer patients; and its levels were closely related to the tumor invasion, clinicopathological characteristics, and survival of PDAC patients.^76^ Besides, Pan et al showed that hsa_circ_0004771 in serum exosomes from early CRC patients was significantly increased.^77^ The fact that the AUC was reached to 0.90 elucidated that exosomal hsa_circ_0004771 could be underlying biomarker to predict early CRC.^77^ In addition, circRNA‐100338 was markedly increased in HCC patients' exosomes.^78^ Its expression was related to DFS and metastases, confirming circRNA‐100338 as a novel HCC biomarker.^78^

## 5 CONCLUSIONS AND FUTURE DIRECTIONS

There is great hope that circRNAs and exo‐circRNAs may be used in medical oncology (Table 1). Our understanding of exosomes has rapidly advanced over the last few years. We look forward to the day of application of circRNAs and exo‐circRNAs in the diagnosis and treatment of GI malignancies.

**TABLE 1:** Overview of identified gastrointestinal malignancy‐associated circRNAs

Type of cancer	CircRNA	Expression	Host gene	Chromosome	Putative function	Potential clinical value	Type of biomarker	Target	Sample detected	References
Gastric cancer	hsa_circ_0000096	Down	HIAT1	Chr1	miRNA sponge	Biomarker	Diagnosis	Not investigated	Tissues	[79]
	hsa_circ_0000190	Down	CNIH4	Chr1	miRNA sponge	Biomarker	Diagnosis	Not investigated	Tissues and plasma	[17]
	hsa_circ_0005654	Down	PRDM5	Chr4	miRNA sponge	Biomarker	Diagnosis	Not investigated	Tissues	[80]
	hsa_circ_0032627 (circDLST)	Up	DLST	Chr14	miRNA sponge	Biomarker	Prognosis	miR‐502‐5p	Tissues	[81]
	hsa_circ_0004339 (circ‐DONSON)	Up	DONSON	Chr21	via NURF complex	Biomarker	Prognosis	Not investigated	Tissues	[51]
	hsa_circ_0002320 (circYAP1)	Down	YAP1	Chr11	miRNA sponge	Biomarker	Prognosis	miR‐367‐5p/p27 Kip1 axis	Tissues	[82]
	hsa_circ_0000199 (circAKT3)	Up	AKT3	Chr1	miRNA sponge	Therapeutic target	Prognosis	miR‐198	Tissues	[49]
	hsa_circ_0081143	Up	COL1A2	Chr7	miRNA sponge	Biomarker	Prognosis	miR‐646	Tissues	[83]
	hsa_circ_0000993	Up	ATL2	Chr2	miRNA sponge	Biomarker	Prognosis	miR‐214‐5p	Tissues	[84]
	hsa_circ_0000140	Down	KIAA0907	Chr1	Not investigated	Biomarker	Diagnosis	Not investigated	Tissues and plasma	[85]
	circLARP4	Up	LARP4	Chr12	miRNA sponge	Biomarker	Prognosis	miR‐424‐5p	Tissues	[86]
	hsa_circ_0130810 (circ‐KIAA1244)	Down	KIAA1244	Chr6	Not investigated	Biomarker	Diagnosis and prognosis	Not investigated	Tissues, plasma and cells	[53]
	hsa_circ_0000745	Down	SPECC1	Chr17	Not investigated	Biomarker	Diagnosis	Not investigated	Tissues, plasma and cells	[87]
	circ‐PSMC3	Down	PSMC3	Chr11	miRNA sponge	Biomarker	Diagnosis and prognosis	miR‐296‐5p	Tissues and cells	[52]
Hepatocellular cancer	hsa_circ_0016788	Up	TRIM11	Chr1	Not investigated	Biomarker	Diagnosis	miR‐486/CDK4 pathway	Tissues and cells	[88]
	hsa_circ_0007874 (circMTO1)	Down	MTO1	Chr6	miRNA sponge	Biomarker and therapeutic target	Diagnosis	miR‐9	Tissues and cells	[56]
	hsa_circ_0001445 (cSMARCA5)	Down	SMARCA5	Chr4	miRNA sponge	Biomarker and therapeutic target	Prognosis	miR‐17‐3p and miR‐181b‐5p	Tissues	[58]
	hsa_circ_0010090 (circFBLIM1)	Up	FBLIM1	Chr1	miRNA sponge	Biomarker	Diagnosis	miR‐346	Tissues and cells	[57]
	circZKSCAN1	Down	ZKSCAN1	Chr7	ceRNA	Biomarker	Diagnosis	Not investigated	Tissues	[89]
	hsa_circ_0000567 (circSETD3)	Down	SETD3	Chr14	miRNA sponge	Biomarker	Prognosis	miR‐421	Tissues and cells	[29]
	hsa_circRNA86 62‐12 (circTRIM33‐12)	Down	TRIM33‐12	Chr1	miRNA sponge	Biomarker	Prognosis	miR‐191	Tissues and cells	[90]
	hsa_circ_0005075	Up	EIF4G3	Chr1	miRNA sponge	Therapeutic target	Diagnosis	miR‐431	Tissues and cells	[91]
	hsa_circ_0072088 (circZFR)	Down	ZFR	Chr5	miRNA sponge	Biomarker	Not investigated	miR‐511	Tissues and cells	[92]
	circSMARCA5	Down	SMARCA5	Chr4	Not investigated	Biomarker	Diagnosis	Not investigated	Tissues, plasma and cells	[93]
	hsa_circ_0000284 (circHIPK3)	Up	HIPK3	Chr11	miRNA sponge	Not investigated	Not investigated	Not investigated	Not investigated	[94]
Colorectal cancer	hsa_circ_0009361	Down	GNB1	Chr1	miRNA sponge	Biomarker	Diagnosis	miR‐582	Tissues and cells	[95]
	hsa_circ_0136666	Up	PRKDC	Chr8	miRNA sponge	Biomarker and therapeutic target	Prognosis	miR‐136	Tissues and cells	[96]
	hsa_circ_0006990 (circVAPA)	Up	VAPA	Chr18	miRNA sponge	Biomarker and therapeutic target	Diagnosis	miR‐101	Tissues and plasma	[60]
	hsa_circ_0055625 (circ_0055625)	Up	DUSP2	Chr2	miRNA sponge	Biomarker	Diagnosis	miR‐106b‐5p	Tissues	[97]
	hsa_circ_0000284 (circHIPK3)	Up	HIPK3	Chr11	miRNA sponge	Biomarker and therapeutic target	Prognosis	miR‐7	Tissues and cells	[62]
	hsa_circ_0142527	Down	PREX2	Chr8	miRNA sponge	Biomarker	Diagnosis	Not investigated	Tissues	[98]
Pancreatic cancer	hsa_circ_0005397 (circRHOT1)	Up	RHOT1	Chr17	miRNA sponge	Therapeutic target	Not investigated	miR‐26b, miR‐125a, miR‐330, and miR‐382	Tissues and cells	[99]

Here, we speculate on two potential future directions for circRNAs and exo‐circRNAs. First, circRNAs and exo‐circRNAs as novel clinical biomarkers of GI malignancies will become the cornerstones of new clinical laboratory analysis methods. By detecting circRNAs and exo‐circRNAs, clinical doctors may diagnose early‐stage GI malignancies, monitor the recurrence and metastasis of GI malignancies, and predict the postoperative survival time. Second, circRNAs and exo‐circRNAs as potential therapeutic targets for GI malignancies may help clinicians extend advanced GI malignancies patients' life and improve their quality of life.

## CONFLICT OF INTEREST

None.
